# Multi-omics and Multi-VOIs to predict esophageal fistula in esophageal cancer patients treated with radiotherapy

**DOI:** 10.1007/s00432-023-05520-5

**Published:** 2024-01-27

**Authors:** Wei Guo, Bing Li, Wencai Xu, Chen Cheng, Chengyu Qiu, Sai-kit Sam, Jiang Zhang, Xinzhi Teng, Lingguang Meng, Xiaoli Zheng, Yuan Wang, Zhaoyang Lou, Ronghu Mao, Hongchang Lei, Yuanpeng Zhang, Ta Zhou, Aijia Li, Jing Cai, Hong Ge

**Affiliations:** 1 Department of Radiation Oncology, The Affiliated Cancer Hospital of Zhengzhou University, Henan Cancer Hospital, 127 Dong Ming Rd, Zhengzhou, Henan Province China; 2 Department of Health Technology and Informatics, The Hong Kong Polytechnic University, Hong Kong SAR, China; 3 Department of Medical Informatics, Nantong University, Nantong, China; 4 School of Electrical and Information Engineering, Jiangsu University of Science and Technology, Zhenjiang, China; 5 Zhengzhou University School of Medicine, Zhengzhou, China

**Keywords:** Esophageal fistula, Esophageal cancer, Radiomics, Dosiomics, Radiotherapy

## Abstract

**Objective:**

This study aimed to develop a prediction model for esophageal fistula (EF) in esophageal cancer (EC) patients treated with intensity-modulated radiation therapy (IMRT), by integrating multi-omics features from multiple volumes of interest (VOIs).

**Methods:**

We retrospectively analyzed pretreatment planning computed tomographic (CT) images, three-dimensional dose distributions, and clinical factors of 287 EC patients. Nine groups of features from different combination of omics [Radiomics (R), Dosiomics (D), and RD (the combination of R and D)], and VOIs [esophagus (ESO), gross tumor volume (GTV), and EG (the combination of ESO and GTV)] were extracted and separately selected by unsupervised (analysis of variance (ANOVA) and Pearson correlation test) and supervised (Student *T* test) approaches. The final model performance was evaluated using five metrics: average area under the receiver-operator-characteristics curve (AUC), accuracy, precision, recall, and F1 score.

**Results:**

For multi-omics using RD features, the model performance in EG model shows: AUC, 0.817 ± 0.031; 95% CI 0.805, 0.825; *p* < 0.001, which is better than single VOI (ESO or GTV).

**Conclusion:**

Integrating multi-omics features from multi-VOIs enables better prediction of EF in EC patients treated with IMRT. The incorporation of dosiomics features can enhance the model performance of the prediction.

**Supplementary Information:**

The online version contains supplementary material available at 10.1007/s00432-023-05520-5.

## Introduction

Esophageal cancer (EC) is a common malignancy worldwide, ranking 7th in incidence and 6th in mortality among reported cancers in 2020 (Curini et al. 2021). Treatment approaches for EC patients include surgery, radiotherapy, and chemotherapy. Despite advances in these treatment regimens, the overall survival rate remains poor (Kelly 2019; Watanabe et al. 2020; Ajani et al. 2019; Ilson 2021; Salmanpour et al. 2023a, b). Most EC patients are diagnosed at an advanced stage, often accompanied by T4, which makes radical esophagectomy almost inapplicable(Takakusagi et al. 2022). Research shows that radiotherapy-based treatment plays an essential role in unresectable EC patients, with concurrent chemoradiotherapy (CCRT) or sequential chemoradiotherapy (SRT) and adjuvant or individual radiotherapy (RT) being common clinical approaches (Kakeji et al. 2021; Mönig et al. 2018; Zhang et al. 2018a).

Esophageal fistula (EF) is a severe complication during radiotherapy treatment with an incidence rate of 4–25% (Sun et al. 2016; Zhu et al. 2020; Zhang et al. 2018b; Tsushima et al. 2016; Hihara et al. 2016). EF can occur at different locations and invade adjacent organs, such as esophageal–mediastinal fistula (EMF), esophagus–respiratory fistula (ERF), and esophagus–aortic fistula (EAF). Once EF occurs during or after treatment, patients often experience poor prognosis with a median survival time of 5–11 months, according to previous reports (Kawakami et al. 2018; Taniyama et al. 2020; Xu et al. 2019; Chen et al. 2021; Pao et al. 2021). Therefore, predicting EF before treatment would help with clinical management to benefit EC patients.

Multiple clinical factors have been discovered to be predictive of EF. For example, Han et al. analyzed the relationship between platelet-to-lymphocyte ratio and EF, achieving a C index of 0.77 (Han et al. 2020). Kawakami et al. investigated several clinical factors, including age, weight loss, T and N stage, lymph node metastasis, radiation prescription dose, and chemotherapy. They found that total circumferential lesion and C-reactive protein ≥ 1.00 mg/dL are risk factors for EF in T4b thoracic esophageal squamous cancer patients treated with definitive CRT (Kawakami et al. 2018). Taniyama et al. analyzed several factors, such as radiation dose, chemotherapy, nutritional support, and tumor information, in NEF patients. They found that large tumor size in the axial plane CT was a risk factor for EF (Taniyama et al. 2020).

With the development of informatics and machine learning techniques, radiomics features extracted from CT images have demonstrated reliable predictive power for radiation toxicity, such as EF (Lambin et al. 2017; Li et al. 2023, 2021; Zhang et al. 2022, 2023). Li et al. collected clinical and radiomics feature from gross tumor volume (GTV) to identify associated risk factors with EF (Li et al. 2023). In addition to hand-crafted radiomic features, deep-learning-based features have been investigated for EF classification. For example, Xu et al. reported improved EF correlation when combining clinical factors with attentional convolutional neural network-based CT features (Xu et al. 2021). Meanwhile, dosiomics features calculated from three-dimensional dose distributions have been proposed by several studies for predicting radiation toxicity (Wu et al. 2020; Liang et al. 2019). However, to our knowledge, none studies have considered either dosiomics features or multi-omics (the combination of radiomics and dosiomics) from the volume of the esophagus in predicting EF.

In this study, we investigated the predictive power of integrating multi-omics features (radiomics and dosiomics, RD) calculated from multiple volumes of interest, including the esophagus (ESO), GTV and the combination of ESO and GTV (EG) for predicting EF. We used nine feature groups from three volumes (ESO, GTV, and EG) and three categories of omics features (R, D, and RD) to compare their predictability in classifying EF.

## Materials and methods

### Patient data

**Fig. 1:**
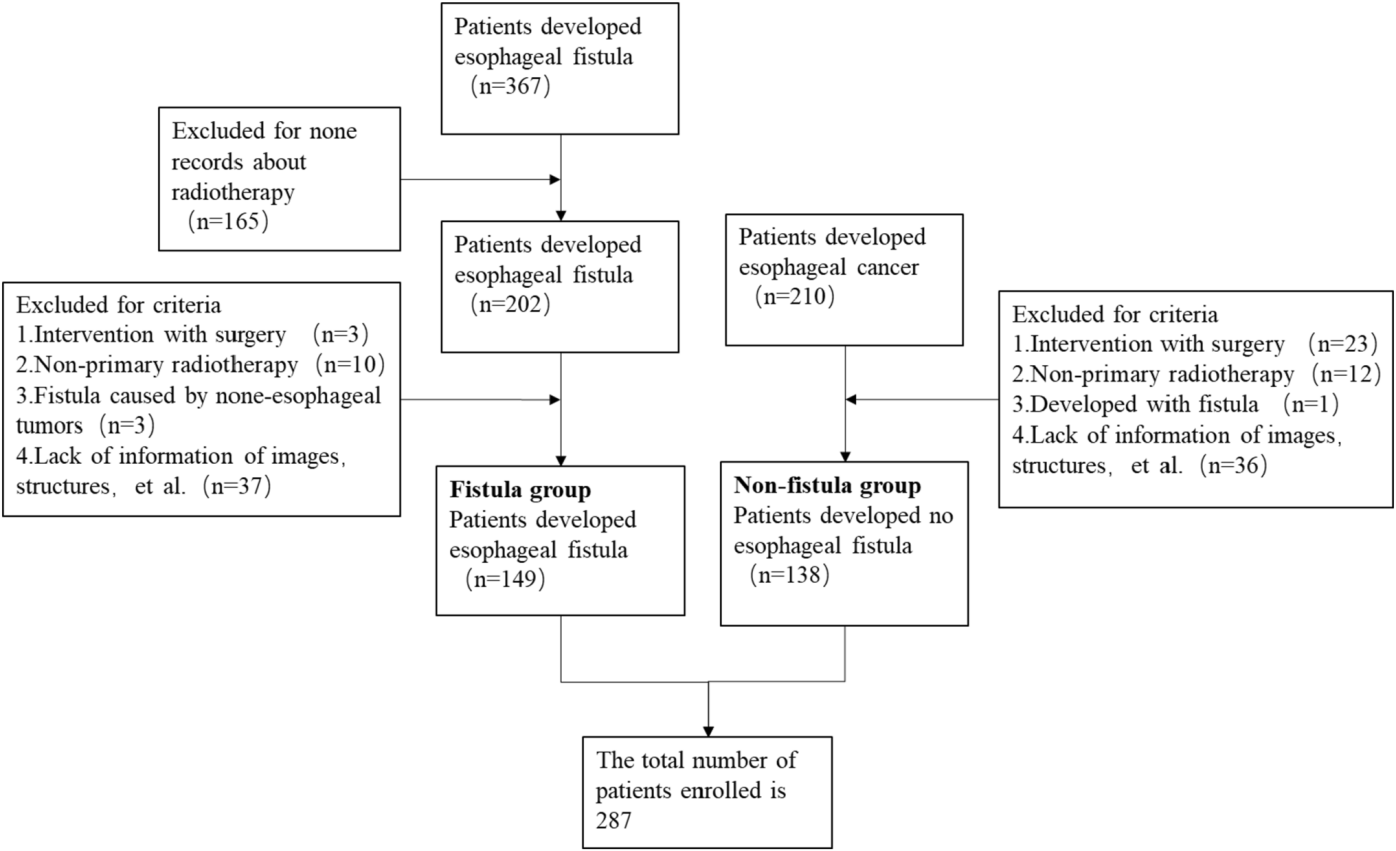
Flowchart of inclusion and exclusion

The study was approved by the Institutional Review Board of the Affiliated Cancer Hospital of Zhengzhou University. 287 EC patients who underwent chemoradiotherapy or radiotherapy were retrospectively analyzed at the Affiliated Cancer Hospital of Zhengzhou University & Henan Cancer Hospital from 2013 to 2022. Among them, 149 EC patients who developed EF were retrieved according to our criteria, and the other 138 EC patients under the criteria without EF were collected randomly, resulting in a 1:1.08 dataset for subsequent analysis. The flow and results of inclusion and exclusion are presented in Fig. 1.

The inclusion criteria involve patients who developed EF were as follows: (1) diagnosed with EC by pathological biopsy; (2) diagnosed with EF through meglumine diatrizoate esophagography or endoscopy with ultrasonography or digestive; (3) received radiotherapy-related treatment using intensity-modulated radiation therapy (IMRT); (4) did not present co-existing tumors of other types at diagnosis; (5) aged 18 or above; and (6) received primary chest radiotherapy. The exclusion criteria were patients: (1) EF was caused by trauma or other invasive tumors; (2) who received surgery during the treatment process; (3) who were diagnosed with pre-existing EF prior to commencement of EC treatment; (4) who suffered from other tumors except EC; and (5) who were diagnosed with esophagitis before start of radiotherapy.

### Data collection

In this study, three types of radiotherapy planning data were used, including planning CT images, radiotherapy planning dose, and contours of organs at risk (OAR) and GTV. The planning CT images were obtained using a Brilliance big bore CT scanner (Philips Electronics, Eindhoven, Netherlands) with consistent scanning parameters (slice thickness 3 mm, tube voltage 120 kV, tube current 360 mA, matrix 512 × 512, a spatial resolution of 1.152 × 1.152 mm). All doses were calculated using the Analytical Anisotropic Algorithm (AAA) from the Eclipse treatment planning system (TPS) with a grid setting of 3 mm. All OARs and GTV were contoured slices by slice by an experienced radiation oncologist with at least 5 years of experience. All image data were exported from the TPS in Digital Imaging and Communications in Medicine (DICOM) format.

Clinical data were collected through the hospital information system authorized by the medical report department, and all data were kept confidential. The demographics included age, gender, and body mass index (BMI). Diagnosis data consisted of pathology, TNM (tumor, node, and metastasis) stage, and lesion location. Three treatment strategies were included in the collected data: CCRT, SRT, and RT. Planning data involved the actual total treatment dose and fractional dose. In the study, CCRT accounted that chemotherapy and radiotherapy proceeded simultaneously; SRT intended that chemotherapy and radiotherapy were given successively; RT was described that only radiotherapy carried out during treatment.

TNM stage was determined by imagological examination using the Union for International Cancer Control TNM (UICC-TNM) classification 7th edition (2009). BMI was calculated using the international standard formula: weight in kilograms divided by height in meters squared. CCRT was defined as receiving chemotherapy and radiotherapy simultaneously, while SRT referred to chemotherapy administered before or after radiotherapy.

### Features extraction

**Fig. 2:**
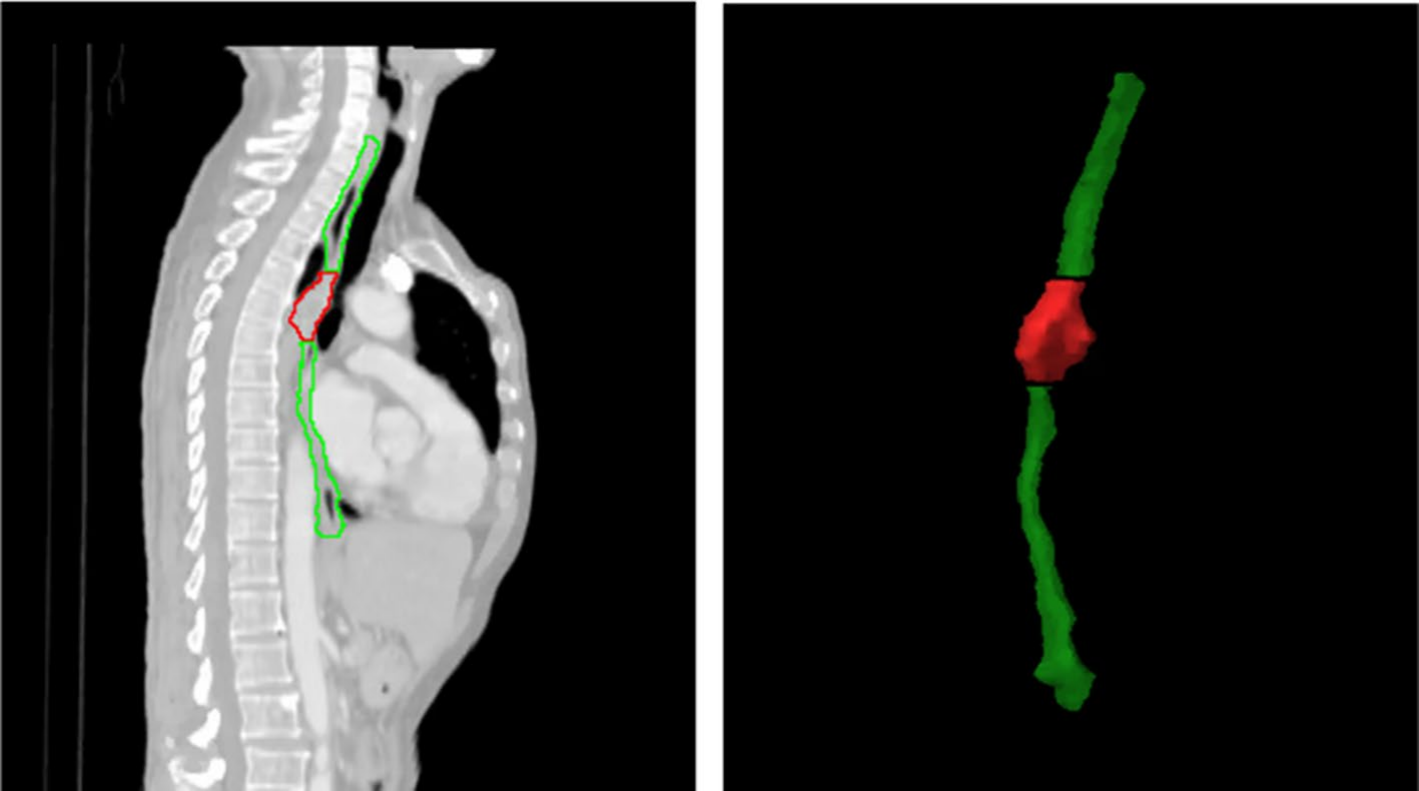
Adopted VOIs in the study: red and green VOIs represent GTV and ESO separately, the combination of above two represent EG

Three volumes of interest (VOI) were adopted in this study, including ESO, GTV, and EG, as shown in Fig. 2. Based on these VOIs, radiomics and dose features were calculated from CT images and dose maps, respectively. More details on these two types of features have been documented in the current body of literature (Lam et al. 2021; Li et al. 2022a, b, c; Zheng et al. 2023).

In this study, a total of 5474 radiomics features were extracted using the Python package pyradiomics, which is a tool for data profiling and quality auditing. These radiomics features were categorized into three types: first-order features (18), three-dimensional shape features (14), and texture features (73). The definitions of these features can be found in the pyradiomics manual (Griethuysen et al. 2017; Zwanenburg et al. 2016) and relevant publications (Lam et al. 2021; Li et al. 2021). A total of 12 images, including original CT images and 11 filtered images, were used to compute the three categories of features. The 11 filtered images included three Laplacian-of-Gaussian filters (sigma = 1, 3, 6 mm) and eight wavelet filters with 63 combinations of high- and low-pass filtering along the three axes. Before feature extraction, a fixed-bin-number gray-level discretization was applied to the original and filtered images with varying bin numbers (10, 20, 30, 40, and 50).

In this study, the dose features were divided into three types: dose-volume histogram (DVH) parameters, scale-invariant 3D dose moments, and dose-based radiomics features. The DVH parameters include Dx and Vx, where Dx represents the dose of x% volume to the whole VOI, and Vx represents the normalized coverage volume by a dose larger than x Gy (Faught et al. 2017). The scale-invariant 3D dose moments represent the dose distributions along the anterior–posterior, medial–lateral, and craniocaudal directions (Buettner et al. 2012; Pham et al. 2011). Except for a constant value for the order of [0, 0, 0], the other 63 dose moments were utilized in the dose features. The dose-based radiomics features were calculated using pyradiomics based on the 3D dose distributions within the VOI, with only the original image being used in the calculation. The resulting 213 dose features were obtained by considering all three types of dose features.

### Feature selection

**Fig. 3:**
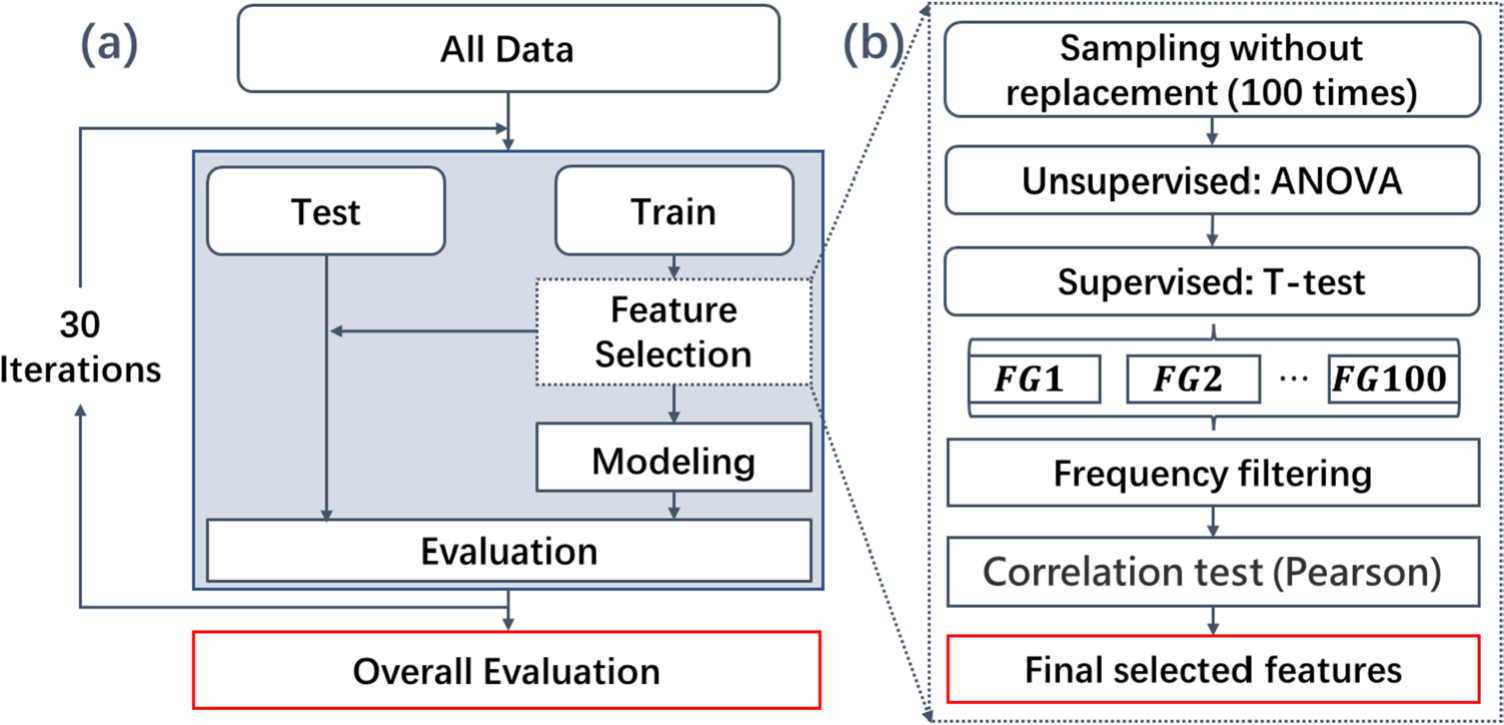
Procedure of the model construction (**a**) and feature selection (**b**)

Before feature selection, all radiomics and dosiomics features were standardized by removing the mean and scaling to unit variance. The feature selection was then performed following the process shown in Fig. 3b. As shown in the Fig. 3, 70% of all data were sampled without replacement to form a training dataset. The training dataset was resampled 100 times to present the different data distributions. For each sampled data, (a) features with zero variance were filtered out using the method of analysis of variance (ANVOA). Then, (b) the rest of features was analyzed with the label of EF using the statistical test of *T* test, and features with a *p* value smaller than 0.01 were kept. After 100 times sampling and the procedure of (a) and (b), one hundred feature groups were obtained. For each preserved feature, the frequency of $i$-th feature was calculated by the equation of ${f}_{i}=\frac{{\sum }_{i}{x}_{i}}{100}$, where $${x}_{i}=\left\{\begin{array}{c}\begin{array}{cc}1,& \text{group with the feature}\\ 0,& \text{group without the feature}\end{array} \\ \end{array}\right.$$. All retained features were sorted based on the frequency. The first 10% (at least 40) of the frequency-sorted features were chosen. The redundancy test further reduced them using the Pearson correlation coefficient with a threshold of 0.5. After that, the reserved features were used to construct a prediction model. The feature combinations, ranging from 1 to the number of reserved features, were adopted. The final feature combination was determined based on the best prediction performance in the testing cohorts.

### Model construction and evaluation

In this study, three feature groups containing dose (D), radiomics (R) features, and the combination of D and R (RD) were used. Finally, the study adopted six feature groups: ESO-D, GTV-D, EG-D, ESO-R, GTV-R, and EG-R. Additionally, the combinations of the volumes of the ESO and GTV for each feature modality, such as ESO-RD, GTV-RD, and EG-RD, were also involved in the study. The choice of using the combination of ESO and GTV as the regions of interest (ROI) is primarily driven by their close anatomical association and proximity to each other. By considering both GTV and Esophagus as the regions of interest, it allows for a comprehensive evaluation of the impact of radiotherapy on the development of esophageal fistula. Therefore, nine models were developed using the aforementioned nine feature groups, following the procedures shown in Fig. 3a.

In the model construction, all data were divided into two groups: the training and testing cohorts with a stratified sampling approach which maintained the same endpoint distribution in two datasets, with a ratio of 7:3. The training cohorts were used for feature selection based on the flowchart of feature selection. The final selected features were used to model the endpoint of the fistula results using the classification algorithm of Ridge through tenfold cross-validation along with automatic hyper-parameter tuning for optimizing the model configuration. The built model was evaluated using five evaluation metrics, including the area under the receiver operating characteristic curve (AUC), precision, accuracy, F1 score, and recall, in both the training and testing cohorts. Based on the aforementioned procedure, different feature combinations were used to construct the classification model, and the final optimal features were determined by the results in the testing cohorts with the largest AUC values.

To assess the prediction power of each feature group, all data were divided into 30 iterations, and the model performance in the training and testing cohorts for each segment was collected to systematically evaluate the model performance in both cohorts. The average and the standard deviation (STD) were computed and presented in the manuscript. The decision curve analysis (DCA) was also employed to assess the model performance by investigating the clinical valuation and avoiding the deficiencies of traditional statistical analyses, for example discrimination and calibration.

### Statistical analysis

The chi-squared and the Kruskal–Wallis tests were used to compare the differences in clinical data between the EF and NEF (None esophageal fistula) groups. The study used a 95% confidence interval (CI) and *p* value, with a *p* < 0.05 indicating statistical significance. The analysis results for all data were performed using SPSS software (version 26) and the Pingouin package of Python (V3.7) (Vallat 2018).

The models mentioned above were compared within each feature group using a two-sided paired student t test. In this study, *p* < 0.05 was also considered researching a significant difference. Furthermore, the DeLong method's 95% CI with 2000 iterations (DeLong et al. 1988) was calculated for all metrics to assess the discrimination ability. Statistical analysis was performed using Python 3.7 and Pingouins 0.5.0 (Vallat 2018).

## Results

### Patients’ characteristics

In this study, a total of 577 patients with EC were screened. Based on the inclusion and exclusion criteria, 149 patients who developed EF and 138 patients developing NEF were eligible for the study. The screening workflow is demonstrated in Fig. 1. The characteristics of the enrolled patients are presented in Table 1. Among patients with EF, 110 (73.8%) developed EMF, while 39 (26.2%) developed ERF. It is worth mentioning that no patient developed more than two kinds of fistula. Among the patients who developed EF, 140 (94%) discontinued treatment immediately, while 9 (6%) continued the treatment schedule after reaching an agreement with their supervising physician. All patients who developed EF were given nutritional support and other symptomatic treatments.

**Table 1:** Characteristics of the clinical information

Clinical factor	Data/*p* value				
	EF		NEF		
Gender (N0., %)					
Male	115	77.18	94	68.12	*p* = 0.085
Female	34	22.82	44	31.88	
Age (N0., %)					
Median	66		66		*p* = 0.027
Range	39–87		46–81		
BMI (N0., %)					
Average	21.42		21.8		*p* = 0.314
Range	13.84–28.73		14.57–32.74		
Pathologic types (N0., %)					
Squamous carcinoma	147		138		*p* = 0.172
Adenocarcinoma	2		0		
T-stage (N0., %)					
T1	0	0	0	0	*p* = 0.68
T2	10	6.72	11	7.97	
T3	105	70.47	101	73.2	
T4	34	22.81	26	18.83	
N-stage (N0., %)					
N0	13	8.72	22	15.94	*p* = 0.133
N1	92	61.75	70	50.72	
N2	39	26.17	38	27.54	
N3	5	3.36	8	5.8	
M-stage (N0., %)					
M0	126	84.56	120	86.96	*p* = 0.563
M1	23	15.44	18	13.04	
Overall-stage (N0., %)					
I	0	0	0	0.00	*p* = 0.307
II	15	10.07	22	15.94	
III	81	54.36	73	52.90	
IV	53	35.57	43	31.16	
Lesion Location (N0., %)					
Cervical	9	6.04	11	7.97	*p* = 0.336
Upper	23	15.44	24	17.39	
Middle	103	69.12	80	57.97	
Lower	14	9.40	23	16.67	
Treatment methods (N0., %)					
CCRT	99	66.44	87	63.04	*p* = 0.457
SRT	23	15.44	18	13.05	
RT	27	18.12	33	23.91	
Fraction dose delivery (N0., %)					
1.8 cGy	78	52.35	106	76.81	*p* < 0.0001
2.0 cGy	71	47.65	32	23.19	

Of the enrolled eligible patients, 209 (72.8%) were male, and 78 (27.2%) were female. The median age was 66 years (range 39–87) in the EF group and 66 years (range 46–81) in the NEF group. EF patients had an average BMI of 21.42 (range 13.84–28.73), while NEF patients had an average BMI of 21.8 (range 14.57–32.74).

Among all patients who underwent pathological diagnosis, only two were diagnosed with adenocarcinoma. None of the 287 patients presented with T1, and the majority of patients developed T3 (70.47% in the EF group, 73.2% in the NEF group). For nodal metastasis, N1 was predominant among patients (61.75% in the EF group, 50.72% in the NEF group). Additionally, 15.44% of patients who developed EF and 13.04% of patients who develop NEF exhibited distant organ or tissue invasion. The overall stage distribution was: EF group, stage I 0%, stage II 10.07%, stage III 54.36%, and stage IV 35.57%; NEF group, stage I 0%, stage II 15.94%, stage III 52.90%, and stage IV 31.16%.

The study involved four types of lesion locations, including cervical, upper, middle, and lower, which were observed in 9, 23, 93, and 14 patients in the EF group and 11, 24, 80, and 23 patients in the NEF group, respectively. Among the 186 patients who received CCRT, platinum-based chemotherapy was administered on the first day of radiotherapy. The chemotherapy regimens were carried out monthly or weekly in the study. SRT was performed, where chemotherapy and radiotherapy were given in succession, regardless of which was initiated first. In the study, 21.02% of patients received radiotherapy alone due to chemotherapy tolerance limits or other reasons.

### Selected feature

After feature selection, a total of 6, 8, and 13 (containing 6 ESO dosiomics, 2 GTV DVH factors and 5 GTV dosiomics features) dose features were chosen in the models using the feature groups of ESO-D, GTV-D and EG-D, respectively, as shown in Supplementary Table 1. Apart from this, there were 35, 24, and 30 (containing 29 ESO radiomics and 1 GTV radiomics features) radiomics features in the final model using features groups of ESO-R, GTV-R, and EG-R, respectively, as shown in Supplementary Table 1. Besides, a total of 29 (including 24 radiomics and 5 dose features), 30 (including 23 radiomics and 7 dose features), and 30 (including 28 ESO radiomics, 1 ESO DVH factor and 1 GTV dosiomics features) features were used in the models using feature groups of ESO-RD, GTV-RD, and EG-RD, respectively, as shown in Supplementary Table 1.

### Model performance

Tables 2 and 3 present the model performance with average values of the evaluating metrics and the corresponding STD. As shown in Table 2, the average AUC ± STD of the model in the training and testing cohorts using dose features for the VOI of ESO, GTV, and EG were 0.732 ± 0.021/0.686 ± 0.049; 0.872 ± 0.015/0.795 ± 0.033; 0.857 ± 0.018/0.777 ± 0.039, respectively. The performances of radiomics-only models were 0.802 ± 0.022/0.774 ± 0.046; 0.802 ± 0.026/0.715 ± 0.043; 0.854 ± 0.027/0.781 ± 0.036. For the cases of multi-omics using RD features, the performance was better than single-omic models with AUCs of 0.835 ± 0.023/0.792 ± 0.045, 0.842 ± 0.016/0.776 ± 0.036, 0.887 ± 0.016/0.817 ± 0.031, respectively.

**Table 2:** Model performance in the training and testing cohorts with average value of the five evaluation metrics for nine classification models using nine feature groups

		ESO-D	GTV-D	EG-D	ESO-R	GTV-R	EG-R	ESO-RD	GTV-RD	EG-RD
AUC	Train	0.732	0.872	0.857	0.802	0.802	0.854	0.835	0.842	0.887
	Test	0.686	0.795	0.777	0.774	0.715	0.781	0.792	0.776	0.817
ACC	Train	0.671	0.795	0.769	0.745	0.743	0.770	0.770	0.774	0.808
	Test	0.623	0.715	0.707	0.717	0.676	0.714	0.737	0.711	0.739
Pre	Train	0.720	0.790	0.767	0.796	0.752	0.802	0.826	0.776	0.835
	Test	0.665	0.716	0.710	0.767	0.688	0.743	0.798	0.718	0.760
Re	Train	0.613	0.829	0.801	0.686	0.756	0.744	0.707	0.797	0.787
	Test	0.570	0.752	0.740	0.658	0.697	0.695	0.667	0.735	0.732
F1	Train	0.657	0.808	0.783	0.736	0.753	0.770	0.761	0.785	0.810
	Test	0.604	0.732	0.724	0.706	0.689	0.716	0.723	0.725	0.744

**Table 3:** Model performance in the training and testing cohorts with 95% CI of the AUC for nine classification models using nine feature groups

		ESO	GTV	EG
D	Train	(0.724, 0.740)	(0.867, 0.877)	(0.850, 0.863)
	Test	(0.667, 0.703)	(0.783, 0.807)	(0.763, 0.791)
R	Train	(0.793, 0.809)	(0.793, 0.811)	(0.843, 0.863)
	Test	(0.757, 0.789)	(0.701, 0.832)	(0.768, 0.794)
RD	Train	(0.827, 0.843)	(0.836, 0.868)	(0.884, 0.894)
	Test	(0.768, 0.794)	(0.775, 0.808)	(0.805, 0.825)

**Fig. 4:**
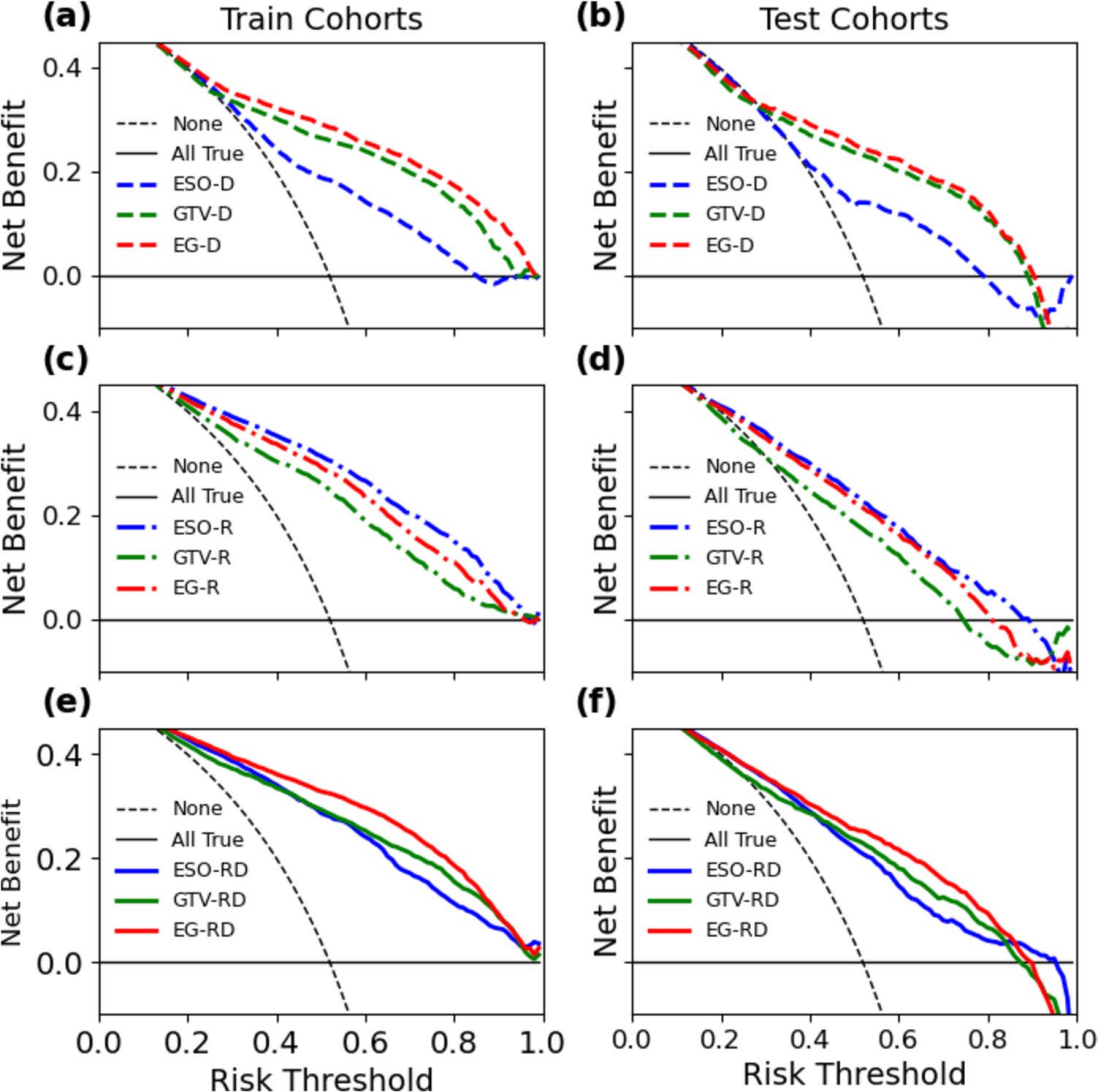
Clinical benefit results for all classification results in training (left) and testing (right) cohorts. **a** and **b** Show models of training and testing for ESO-D, GTV-D, and EG-D. **c** and **d** Show models of training and testing for ESO-R, GTV-R, and EG-R. **e** and **f** Show models of training and testing for ESO-RD, GTV-RD, and EG-RD. The different models were represented by three different color lines of blue, green and red, which were drawn by dashed, dot-dashed and solid types respectively. The dashed and solid black lines represents the net benefit of all patients suffering EF and NEF

In Fig. 4, we present the results of our clinical benefit analysis. Across a wide range of risk thresholds, the dosiomics group showed that the EG-D model outperformed the ESO-D and GTV-D models in terms of clinical benefits. We observed a similar trend in the multi-omics group (i.e., RD), where the EG-RD model achieved higher clinical benefits than the ESO-RD and GTV-RD models in most risk thresholds. However, for the radiomics group, we found that the ESO-R model provided higher clinical benefits than the GTV-R and EG-R models for most risk thresholds (Fig. 4).

## Discussion

This study aimed to develop an effective prediction model for EF in EC patients following treatment. The novelty lies in the use of stand-alone and different combinations of multi-omics features (R, D, RD) from multiple VOIs (ESO, GTV, and EG) for prediction model development. The results showed that the model using multi-omics from multi-VOIs, i.e., EG-RD, obtained the best classification results (*p* < 0.05) with an average AUC of 0.817 in the testing cohorts for prognosing EF for EC patients receiving radiotherapy, compared to the other models. These findings suggest that radiation toxicity of EF would be predicted more accurately using multi-omics features from multi-VOIs (i.e., EG-RD).

The EG-RD model enables more accurate individualized fistula risk prediction, aiding identification of high-risk patients for alternative treatments through counseling incorporating quantitative risks. Personalized adaptations may also be informed by predictive analytics, such as intensifying nutrition support or restricting doses to highlighted regions. Treatment planning could optimize dose distributions considering each patient’s toxicity risks by leveraging the predictive model. Collecting multi-region omics data advances precision oncology, proactively mitigating complications to ultimately improve patient outcomes (Rezaeijo et al. 2022; Whybra et al. 2019; Jahangirimehr et al. 2022).

After selecting features, the best model using EG-RD features only contained two dose features: D0.8 of the esophagus and one dosiomics feature (original_glcm_Imc1_1.00) from GTV. This indicates that the dose feature is correlated with EF and can improve EF prediction when combined with radiomics features. Among the 29 selected features, 28 features are from the ESO, which may imply that the ESO has a stronger correlation with EF compared to the GTV. However, the GTV-D model achieved a relatively high AUC value (AUC 0.795) compared to the GTV-R model with an AUC of 0.715. All above findings indicate that the GTV is an essential VOI correlated with EF. Furthermore, the radiomics features played a dominant role in the EG-RD model compared to the dose features, with a feature number of 28 to 2. Regarding clinical information, only fraction dose delivery showed significant correlation with EF incidence with *p* < 0.05, as shown in Table 1. This result is inconsistent with previous research findings with high predictability (see Table 4) may be caused by the discrepancy in data distribution.

**Table 4:** Literature summary for predicting EF treating by radiotherapy

Reference	Patients Size	VOI	Features	Performance
Li et al. (2023)	172 (43 EF)	GTV	Clinical and radiomics	AUC 0.896
Xu et al. (2021)	558 (186 EF)	GTV and anatomical surrounding	Clinical and radiomics	C-index 0.901
Zhu et al. (2021)	1653 (76 EF)	GTV	Clinical and radiomics	C-index 0.831
Shi et al. (2022)	204 (54 EF)	GTV	Quantitative CT factors	AUC 0.917
Xu et al. (2019)	408 (136 EF)	-	Clinical	C-index 0.805
Gui et al. (2022)	324 (81 EF)	GTV	Clinical and CT factors	AUC 0.848

Regarding the model performance, the highest testing AUC (average value) is 0.817, which is lower than previous studies (Li et al. 2023; Shi et al. 2022; Gui et al. 2022). This may be due to two reasons. First, the analysis methods are different, with multiple dividing used in our study compared to training on a single train-test splitting method used in the other studies. Second, the dataset distribution is almost balanced in our study (NEF to EF ratio of about 1:1), while it is unbalanced in the other studies (NEF to EF ratio of n:1, where *n* > 2). Unbalanced data can cause over-fitting or under-fitting problems and affect the model’s generalization ability.

**Fig. 5:**
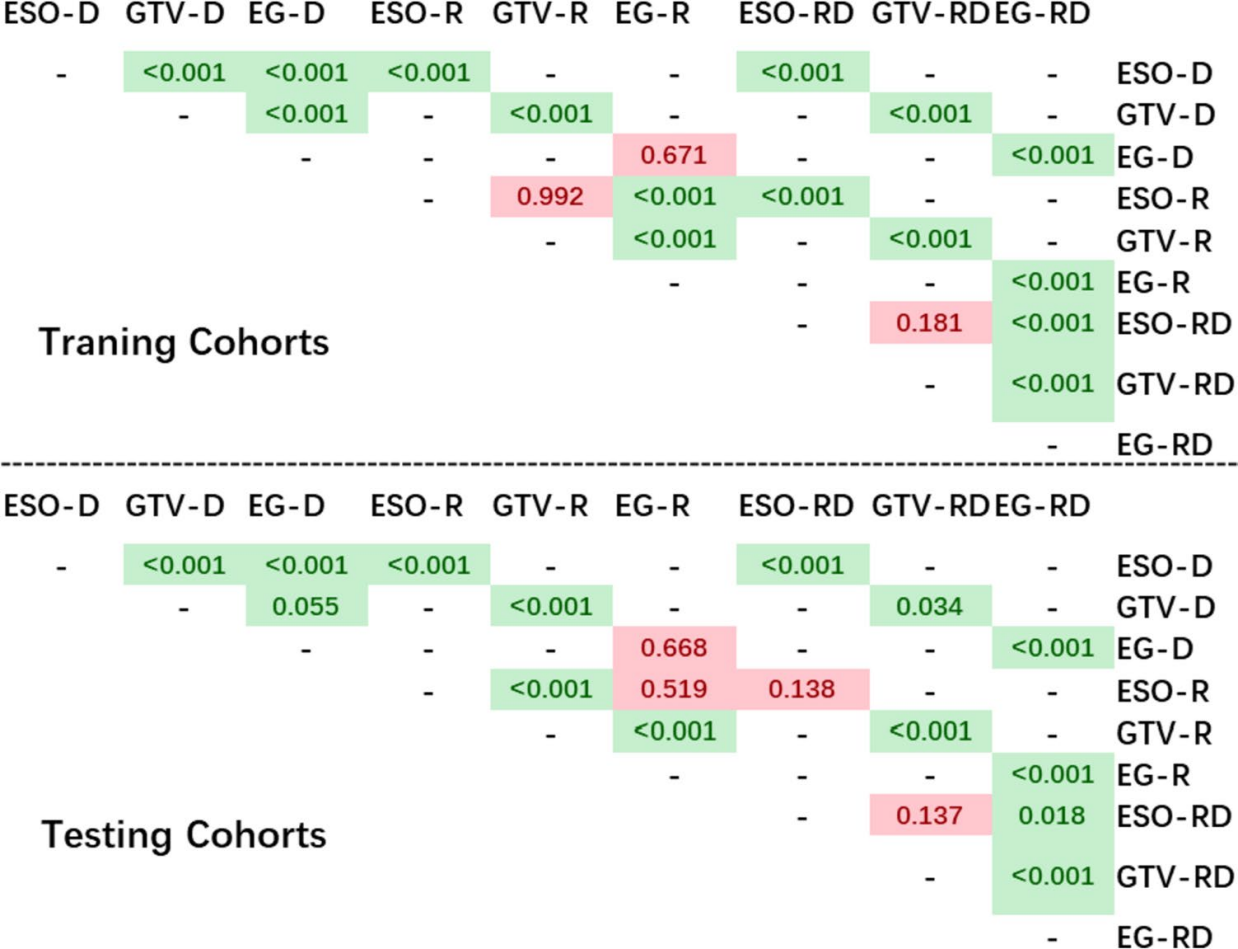
Statistical analysis between classification models

Figure 5 shows the statistical analysis of all models. As shown in the figure, in the model using dose features, the EG-D model’s performance achieved a significantly higher AUC value compared to the ESO-D model in both the training and testing cohorts (*p* < 0.001). While the EG-D model's AUC value was significantly lower than the GTV-D model in the training cohorts (AUC 0.857 to 0.872, *p* < 0.001), but was no significant difference was found in the testing cohorts (*p* value = 0.055), and the GTV-D model performed better than ESO-D model both in the training and testing cohorts with *p* < 0.001. This suggests that the dose features from multiple VOIs were unable to improve the prediction performance, and the VOI of GTV had a stronger predictive power than ESO. However, the situation was reversed for the radiomics feature group. For radiomics, the ESO-R and GTV-R models' performance had no significant difference in the training cohorts (*p* = 0.992), but the ESO-R model achieved significantly better classification results in testing than the GTV-R model with *p* < 0.001. On the contrary, the EG-R model obtained a significantly higher AUC value than the ESO-R model (*p* < 0.001), but there was no significant difference in the testing cohorts with a p value of 0.519. In the multi-omics group, there were no significant differences in both the training (*p* = 0.181) and testing (*p* = 0.137) cohorts for the two models using ESO-RD and GTV-RD models. The EG-RD model obtained significantly better prediction results than the ESO-RD and GTV-RD models in both the training and testing cohorts, with *p* values of *p* < 0.001/*p* < 0.001 and *p* < 0.001/*p* < 0.05, respectively. These findings suggest that multi-omics features from multi-VOIs can achieve a higher correlation with EF than a single VOI of ESO or GTV.

As shown in Fig. 5, when comparing different VOIs, radiomics-based models using ESO-R and GTV-R have a stronger prediction ability than dosiomics-based models of ESO-D and GTV-D in both training and testing cohorts (*p* < 0.001), respectively. However, there is no significant difference between the EG-R and EG-D models in both training and testing cohorts, with *p* values of 0.671 and 0.668, respectively. When comparing RD-based models and D-based models, the RD-based models (ESO-RD, GTV-RD, and EG-RD) can achieve better classification results than D-based models (ESO-D, GTV-D, and EG-D) in both training and testing cohorts with all *p* values < 0.05, respectively. Except for the VOI of ESO in the testing cohorts (ESO-RD to ESO-R, *p* = 0.138 in testing cohorts), the RD-based models (ESO-RD, GTV-RD, and EG-RD) have significantly better prediction results than R-based models (ESO-R, GTV-R, and EG-RD) with *p* < 0.001, respectively.

Although we carried out a comprehensive analysis among VOI and feature groups for predicting EF in EC patients treated with radiotherapy alone or in combination with chemotherapy, there are still some limitations in our study. First, our dataset was collected solely from our institution and did not include data from other centers. The generalizability of the presented results may be weakened by the use of single-center data. Therefore, a multi-center and larger dataset should be used to investigate the performance of using multi-omics features from multi-VOIs and the model's generalization and robustness. Second, the patients included in our study received radiotherapy using IMRT technique. The investigation should be reanalyzed for other radiotherapy techniques, such as Volume Modulated Radiation Therapy (VMAT) or a combination of IMRT and VMAT owing to the difference in dose distribution. The use of homogeneous IMRT treatment may undermine the generalizability of the results, potentially impacting their overall quality. Third, several studies have analyzed feature repeatability and reproducibility by considering the impact of VOI segmentation and CT image acquisition (Teng et al. 2022a, b; Placidi et al. 2020; Zwanenburg et al. 2019; Larue et al. 2017; Lafata et al. 2018). It is worth comprehensively studying models that use robust and repeatable features before clinical use.

## Conclusion

In this study, we investigated the predictive power of multi-omics and single-omic features from the VOI of the esophagus and/or GTV, as well as their combination in predicting EF for EC patients treated with IMRT radiotherapy. Integrating multi-omics features from multi-VOIs enables accurate prediction of EF in EC patients treated with (chemo)-radiotherapy of IMRT. The incorporation of dose features can enhance the performance of the prediction model.

## Supplementary Information

Below is the link to the electronic supplementary material.

Supplementary file1 (DOCX 38 KB)

## Data Availability

The datasets used for this study are available on request to the corresponding author.
