# Supplementary material for: Multi-omics and Multi-VOIs to predict esophageal fistula in esophageal cancer patients treated with radiotherapy
Source: J Cancer Res Clin Oncol. 2024 Jan 27;150(2):39. doi: 10.1007/s00432-023-05520-5 (PMC10821966; doi:10.1007/s00432-023-05520-5)
Supplement: Supplementary file 1 — Supplementary file1 (DOCX 38 KB) [file 432_2023_5520_MOESM1_ESM.docx]

*Supplementary Table 1 The selected features of three features sets (R, D, and RD) for VOIS of ESO, GTV and EG..*

| Name | Features |
| --- | --- |
| ESO-D | ESO_V0.94  ESO_dosiomics_original_glcm_Autocorrelation_1.00_binWidth  ESO_D0.80_Gy  ESO_dosiomics _original_glcm_MaximumProbability_1.00_binWidth  ESO_dosiomics _original_glcm_Idn_1.00_binWidth  ESO_dosiomics _original_glcm_DifferenceVariance_1.00_binWidth |
| ESO-R | ESO_radiomics_wavelet-HLL_glrlm_HighGrayLevelRunEmphasis_30_binCount  ESO_radiomics_wavelet-HLH_glrlm_RunLengthNonUniformity_10_binCount  ESO_radiomics_wavelet-HLH_glszm_SizeZoneNonUniformityNormalized_20_binCount  ESO_radiomics_wavelet-HLH_glszm_SmallAreaLowGrayLevelEmphasis_10_binCount  ESO_radiomics_wavelet-HLH_glszm_GrayLevelNonUniformity_10_binCount  ESO_radiomics_wavelet-LHL_firstorder_Mean_10_binCount  ESO_radiomics_wavelet-LLH_gldm_DependenceVariance_10_binCount  ESO_radiomics_wavelet-LHL_firstorder_Skewness_10_binCount  ESO_radiomics_wavelet-HHL_firstorder_Mean_10_binCount  ESO_radiomics_wavelet-HLL_glcm_Correlation_10_binCount  ESO_radiomics_wavelet-LLH_firstorder_Minimum_20_binCount  ESO_radiomics_log-sigma-6-0-mm-3D_ngtdm_Contrast_50_binCount  ESO_radiomics_wavelet-LLH_glszm_SizeZoneNonUniformity_10_binCount  ESO_radiomics_wavelet-LLH_glszm_SmallAreaEmphasis_10_binCount  ESO_radiomics_wavelet-LLH_glszm_SmallAreaLowGrayLevelEmphasis_50_binCount  ESO_radiomics_wavelet-LLH_glcm_ClusterShade_40_binCount  ESO_radiomics_log-sigma-6-0-mm-3D_glcm_Imc1_20_binCount  ESO_radiomics_log-sigma-6-0-mm-3D_firstorder_Skewness_50_binCount  ESO_radiomics_wavelet-LHH_glrlm_LongRunLowGrayLevelEmphasis_30_binCount  ESO_radiomics_log-sigma-6-0-mm-3D_glszm_SmallAreaLowGrayLevelEmphasis_50_binCount  ESO_radiomics_wavelet-HLL_glszm_SizeZoneNonUniformityNormalized_10_binCount  ESO_radiomics_wavelet-HLL_glszm_GrayLevelNonUniformity_10_binCount  ESO_radiomics_wavelet-HLL_glrlm_RunVariance_50_binCount  ESO_radiomics_wavelet-LHH_glszm_SizeZoneNonUniformityNormalized_10_binCount  ESO_radiomics_log-sigma-6-0-mm-3D_glszm_SmallAreaEmphasis_40_binCount  ESO_radiomics_log-sigma-6-0-mm-3D_glszm_SizeZoneNonUniformity_50_binCount  ESO_radiomics_log-sigma-6-0-mm-3D_gldm_DependenceVariance_20_binCount  ESO_radiomics_wavelet-HHL_glszm_SmallAreaEmphasis_10_binCount  ESO_radiomics_log-sigma-6-0-mm-3D_glszm_ZoneEntropy_20_binCount  ESO_radiomics_log-sigma-6-0-mm-3D_glrlm_ShortRunLowGrayLevelEmphasis_50_binCount  ESO_radiomics_wavelet-HHL_firstorder_Variance_40_binCount  ESO_radiomics_log-sigma-6-0-mm-3D_ngtdm_Strength_20_binCount  ESO_radiomics_log-sigma-6-0-mm-3D_gldm_DependenceEntropy_40_binCount  ESO_radiomics_wavelet-HHL_glcm_ClusterShade_10_binCount  ESO_radiomics_wavelet-LLH_glcm_Correlation_40_binCount |
| ESO-RD | ESO_radiomics_wavelet-HHL_firstorder_Variance_40_binCount  ESO_radiomics_wavelet-HHL_glcm_ClusterShade_10_binCount  ESO_radiomics_wavelet-LHL_firstorder_Skewness_10_binCount  ESO_radiomics_wavelet-LLH_gldm_DependenceVariance_10_binCount  ESO_radiomics_wavelet-LLH_glszm_SmallAreaLowGrayLevelEmphasis_50_binCount  ESO_radiomics_wavelet-LLH_glszm_SmallAreaEmphasis_10_binCount  ESO_radiomics_wavelet-LLH_glszm_SizeZoneNonUniformity_10_binCount  ESO_radiomics_wavelet-LLH_glcm_Correlation_40_binCount  ESO_radiomics_wavelet-LLH_glcm_ClusterShade_40_binCount  ESO_radiomics_wavelet-LLH_firstorder_Minimum_20_binCount  ESO_radiomics_log-sigma-6-0-mm-3D_ngtdm_Contrast_50_binCount  ESO_radiomics_log-sigma-6-0-mm-3D_ngtdm_Strength_20_binCount  ESO_radiomics_log-sigma-6-0-mm-3D_gldm_DependenceEntropy_40_binCount  ESO_radiomics_log-sigma-6-0-mm-3D_gldm_DependenceVariance_20_binCount  ESO_radiomics_log-sigma-6-0-mm-3D_glszm_SmallAreaLowGrayLevelEmphasis_50_binCount  ESO_radiomics_log-sigma-6-0-mm-3D_glszm_SizeZoneNonUniformity_50_binCount  ESO_radiomics_log-sigma-6-0-mm-3D_glszm_SmallAreaEmphasis_40_binCount  ESO_radiomics_log-sigma-6-0-mm-3D_glszm_ZoneEntropy_20_binCount  ESO_radiomics_log-sigma-6-0-mm-3D_glrlm_ShortRunLowGrayLevelEmphasis_50_binCount  ESO_radiomics_log-sigma-6-0-mm-3D_glcm_Imc1_20_binCount  ESO_radiomics_wavelet-LHL_firstorder_Mean_10_binCount  ESO_radiomics_wavelet-LHH_glrlm_LongRunLowGrayLevelEmphasis_30_binCount  ESO_dosiomics_original_glcm_Idn_1.00_binWidth  ESO_radiomics_wavelet-LHH_glszm_SizeZoneNonUniformityNormalized_10_binCount  ESO_dosiomics_original_glcm_DifferenceVariance_1.00_binWidth  ESO_dosiomics_original_glcm_Autocorrelation_1.00_binWidth  ESO_DVH_V0.94  ESO_DVH_D0.80_Gy  ESO_radiomics_wavelet-HHL_glszm_SmallAreaEmphasis_10_binCount |
| GTV-D | GTV_DVH_V50.00Gy  GTV_DVH_V0.99  GTV_dosiomics _original_glcm_Imc1_1.00_binWidth  GTV_dosiomics _original_glcm_Idn_1.00_binWidth  GTV_dosiomics _original_glcm_Contrast_1.00_binWidth  GTV_dosiomics _original_glcm_Autocorrelation_1.00_binWidth  GTV_dosiomics_original_firstorder_Skewness_1.00_binWidth  GTV_DVH_V64.00Gy |
| GTV-R | GTV_radiomics_wavelet-LHH_gldm_DependenceNonUniformityNormalized_50_binCount  GTV_ radiomics _wavelet-LHH_gldm_HighGrayLevelEmphasis_50_binCount  GTV_ radiomics _wavelet-LHL_glszm_GrayLevelVariance_40_binCount  GTV_ radiomics _wavelet-LHL_glszm_SizeZoneNonUniformity_30_binCount  GTV_ radiomics _wavelet-LHL_gldm_DependenceNonUniformityNormalized_30_binCount  GTV_ radiomics _wavelet-LLH_glrlm_RunEntropy_50_binCount  GTV_ radiomics _wavelet-LLH_glrlm_LongRunLowGrayLevelEmphasis_50_binCount  GTV_ radiomics _wavelet-LLH_glrlm_RunEntropy_10_binCount  GTV_ radiomics _wavelet-LLH_glcm_MaximumProbability_20_binCount  GTV_ radiomics _wavelet-LLH_glcm_Imc1_20_binCount  GTV_ radiomics _wavelet-LLH_firstorder_Skewness_40_binCount  GTV_ radiomics _wavelet-LLH_glcm_InverseVariance_30_binCount  GTV_ radiomics _wavelet-LHL_glcm_ClusterShade_10_binCount  GTV_ radiomics _wavelet-LHL_glcm_Autocorrelation_10_binCount  GTV_ radiomics _wavelet-LHL_firstorder_Variance_40_binCount  GTV_ radiomics _wavelet-LLH_glszm_LowGrayLevelZoneEmphasis_50_binCount  GTV_ radiomics _wavelet-LLH_gldm_DependenceVariance_50_binCount  GTV_ radiomics _wavelet-HLL_glcm_ClusterShade_30_binCount  GTV_ radiomics _wavelet-HLL_firstorder_Maximum_40_binCount  GTV_ radiomics _wavelet-HLL_glcm_InverseVariance_20_binCount  GTV_ radiomics _wavelet-HLL_glcm_Correlation_20_binCount  GTV_ radiomics _wavelet-HLL_firstorder_RootMeanSquared_30_binCount  GTV_ radiomics _wavelet-LHH_ngtdm_Coarseness_30_binCount  GTV_ radiomics _wavelet-LHH_firstorder_Uniformity_20_binCount |
| GTV-RD | GTV_dosiomics _original_glcm_Autocorrelation_1.00_binWidth  GTV_dosiomics _original_firstorder_Skewness_1.00_binWidth  GTV_dosiomics _original_glcm_Idn_1.00_binWidth  GTV_radiomics_wavelet-LLH_glcm_Imc1_20_binCount  GTV_radiomics_wavelet-LLH_glcm_MaximumProbability_20_binCount  GTV_radiomics_wavelet-LLH_glcm_InverseVariance_30_binCount  GTV_radiomics_wavelet-LLH_glrlm_RunEntropy_10_binCount  GTV_radiomics_wavelet-LLH_glrlm_LongRunLowGrayLevelEmphasis_50_binCount  GTV_radiomics_wavelet-LLH_glrlm_RunEntropy_50_binCount  GTV_radiomics_wavelet-LLH_glszm_LowGrayLevelZoneEmphasis_50_binCount  GTV_radiomics_wavelet-LLH_gldm_DependenceVariance_50_binCount  GTV_radiomics_wavelet-LHL_firstorder_Variance_40_binCount  GTV_radiomics_wavelet-LHL_glcm_Autocorrelation_10_binCount  GTV_radiomics_wavelet-LHL_glcm_ClusterShade_10_binCount  GTV_radiomics_wavelet-LHL_glszm_SizeZoneNonUniformity_30_binCount  GTV_radiomics_wavelet-LHL_glszm_GrayLevelVariance_40_binCount  GTV_radiomics_wavelet-LHL_gldm_DependenceNonUniformityNormalized_30_binCount  GTV_radiomics_wavelet-LHH_firstorder_Uniformity_20_binCount  GTV_radiomics_wavelet-LHH_gldm_DependenceNonUniformityNormalized_50_binCount  GTV_radiomics_wavelet-LHH_gldm_HighGrayLevelEmphasis_50_binCount  GTV_radiomics_wavelet-LHH_ngtdm_Coarseness_30_binCount  GTV_radiomics_wavelet-HLL_firstorder_RootMeanSquared_30_binCount  GTV_radiomics_wavelet-HLL_firstorder_Maximum_40_binCount  GTV_radiomics_wavelet-HLL_glcm_Correlation_20_binCount  GTV_radiomics_wavelet-HLL_glcm_InverseVariance_20_binCount  GTV_radiomics_wavelet-HLL_glcm_ClusterShade_30_binCount  GTV_DVH_V0.99  GTV_DVH_V50.00Gy  GTV_DVH_V64.00Gy  GTV_dosiomics_GTVZ_original_glcm_Imc1_1.00_binWidth |
| EG-D | ESO_dosiomics_EsophagusGTV_D0.80_Gy  ESO_dosiomics_EsophagusGTV_V0.94  ESO_dosiomics_EsophagusGTV_original_glcm_Autocorrelation_1.00_binWidth  ESO_dosiomics_EsophagusGTV_original_glcm_DifferenceVariance_1.00_binWidth  ESO_dosiomics_EsophagusGTV_original_glcm_Idn_1.00_binWidth  ESO_dosiomics_EsophagusGTV_original_glcm_MaximumProbability_1.00_binWidth  GTV_DVH_V0.99  GTV_DVH_V64.00Gy  GTV_dosiomics_original_firstorder_Skewness_1.00_binWidth  GTV_dosiomics_original_glcm_Autocorrelation_1.00_binWidth  GTV_dosiomics_original_glcm_Contrast_1.00_binWidth  GTV_dosiomics_original_glcm_Idn_1.00_binWidth  GTV_dosiomics_original_glcm_Imc1_1.00_binWidth |
| EG-R | ESO_radiomics_wavelet-HLH_glszm_SmallAreaLowGrayLevelEmphasis_10_binCount  ESO_radiomics_wavelet-HLH_glrlm_RunLengthNonUniformity_10_binCount  ESO_radiomics_wavelet-LLH_glszm_SizeZoneNonUniformity_10_binCount  ESO_radiomics_wavelet-HLL_glszm_SizeZoneNonUniformityNormalized_10_binCount  ESO_radiomics_wavelet-HLL_glszm_GrayLevelNonUniformity_10_binCount  ESO_radiomics_wavelet-HLL_glrlm_RunVariance_50_binCount  ESO_radiomics_wavelet-HLL_glrlm_HighGrayLevelRunEmphasis_30_binCount  ESO_radiomics_wavelet-HLL_glcm_Correlation_10_binCount  ESO_radiomics_wavelet-LHH_glszm_SizeZoneNonUniformityNormalized_10_binCount  ESO_radiomics_wavelet-LHH_glrlm_LongRunLowGrayLevelEmphasis_30_binCount  ESO_radiomics_wavelet-LHL_firstorder_Skewness_10_binCount  ESO_radiomics_wavelet-LHL_firstorder_Mean_10_binCount  ESO_radiomics_wavelet-LLH_gldm_DependenceVariance_10_binCount  ESO_radiomics_wavelet-LLH_glszm_SmallAreaLowGrayLevelEmphasis_50_binCount  ESO_radiomics_wavelet-LLH_glszm_SmallAreaEmphasis_10_binCount  ESO_radiomics_wavelet-LLH_glcm_Correlation_40_binCount  ESO_radiomics_wavelet-HLH_glszm_GrayLevelNonUniformity_10_binCount  ESO_radiomics_wavelet-LLH_glcm_ClusterShade_40_binCount  ESO_radiomics_wavelet-LLH_firstorder_Minimum_20_binCount  ESO_radiomics_log-sigma-6-0-mm-3D_ngtdm_Contrast_50_binCount  ESO_radiomics_log-sigma-6-0-mm-3D_ngtdm_Strength_20_binCount  ESO_radiomics_log-sigma-6-0-mm-3D_gldm_DependenceEntropy_40_binCount  ESO_radiomics_log-sigma-6-0-mm-3D_gldm_DependenceVariance_20_binCount  ESO_radiomics_log-sigma-6-0-mm-3D_glszm_SmallAreaLowGrayLevelEmphasis_50_binCount  ESO_radiomics_log-sigma-6-0-mm-3D_glszm_SizeZoneNonUniformity_50_binCount  ESO_radiomics_log-sigma-6-0-mm-3D_glszm_SmallAreaEmphasis_40_binCount  ESO_radiomics_log-sigma-6-0-mm-3D_glszm_ZoneEntropy_20_binCount  ESO_radiomics_log-sigma-6-0-mm-3D_glrlm_ShortRunLowGrayLevelEmphasis_50_binCount  ESO_radiomics_log-sigma-6-0-mm-3D_glcm_Imc1_20_binCount  GTV_radiomics_wavelet-HLL_glcm_ClusterShade_30_binCount |
| EG-RD | ESO_radiomics_log-sigma-6-0-mm-3D_ngtdm_Strength_20_binCount  ESO_radiomics_wavelet-LLH_glszm_SmallAreaEmphasis_10_binCount  ESO_DVH_D0.80_Gy  ESO_radiomics_wavelet-LHL_firstorder_Skewness_10_binCount  ESO_radiomics_wavelet-LHH_glrlm_LongRunLowGrayLevelEmphasis_30_binCount  ESO_radiomics_wavelet-LHH_glszm_SizeZoneNonUniformityNormalized_10_binCount  ESO_radiomics_wavelet-HLL_glcm_Correlation_10_binCount  ESO_radiomics_wavelet-HLL_glrlm_HighGrayLevelRunEmphasis_30_binCount  ESO_radiomics_wavelet-HLL_glrlm_RunVariance_50_binCount  ESO_radiomics_wavelet-HLL_glszm_GrayLevelNonUniformity_10_binCount  ESO_radiomics_wavelet-HLL_glszm_SizeZoneNonUniformityNormalized_10_binCount  ESO_radiomics_wavelet-HLH_glrlm_RunLengthNonUniformity_10_binCount  ESO_radiomics_wavelet-HLH_glszm_GrayLevelNonUniformity_10_binCount  ESO_radiomics_wavelet-HLH_glszm_SmallAreaLowGrayLevelEmphasis_10_binCount  ESO_radiomics_wavelet-HLH_glszm_SizeZoneNonUniformityNormalized_20_binCount  ESO_radiomics_wavelet-HHL_firstorder_Mean_10_binCount  ESO_radiomics_wavelet-HHL_firstorder_Variance_40_binCount  ESO_radiomics_wavelet-HHL_glcm_ClusterShade_10_binCount  ESO_radiomics_wavelet-LHL_firstorder_Mean_10_binCount  ESO_radiomics_wavelet-LLH_gldm_DependenceVariance_10_binCount  ESO_radiomics_wavelet-LLH_glszm_SmallAreaLowGrayLevelEmphasis_50_binCount  ESO_radiomics_log-sigma-6-0-mm-3D_gldm_DependenceVariance_20_binCount  ESO_radiomics_log-sigma-6-0-mm-3D_glcm_Imc1_20_binCount  ESO_radiomics_log-sigma-6-0-mm-3D_glrlm_ShortRunLowGrayLevelEmphasis_50_binCount  ESO_radiomics_log-sigma-6-0-mm-3D_glszm_ZoneEntropy_20_binCount  ESO_radiomics_log-sigma-6-0-mm-3D_glszm_SmallAreaEmphasis_40_binCount  ESO_radiomics_log-sigma-6-0-mm-3D_glszm_SizeZoneNonUniformity_50_binCount  ESO_radiomics_log-sigma-6-0-mm-3D_glszm_SmallAreaLowGrayLevelEmphasis_50_binCount  ESO_radiomics_log-sigma-6-0-mm-3D_gldm_DependenceEntropy_40_binCount  GTV_dosiomics_original_glcm_Imc1_1.00_binWidth |

*Supplementary Table 2 The standard-deviation of the model performance in the training and testing cohorts for nine models. The color is greenner, the values is lower.*
